# 835. Improvement in Diet Attenuates Antiretroviral Therapy (ART) Associated Weight Gain in Persons with Human Immunodeficiency Virus (PWH)

**DOI:** 10.1093/ofid/ofab466.1031

**Published:** 2021-12-04

**Authors:** Yesha Patel, Anjali Doshi, Anna Levesque, Shelsie Lindor, Robert Moranville, Sheila Okere, Danielle Robinson, Lauren Taylor, Mark Lustberg, Carlos Malvestutto

**Affiliations:** 1 The Ohio State University Wexner Medical Center, Columbus, Ohio; 2 The Ohio State University College of Medicine, Columbus, Ohio; 3 The Ohio State University, Columbus, Ohio

## Abstract

**Background:**

Weight gain among PWH on ART is a growing clinical concern. We explore factors associated with weight gain at The Ohio State University Wexner Medical Center Infectious Diseases Clinic.

**Methods:**

This was a single-center, retrospective, cohort study of adult PWH on ART for at least 3 months seen at our clinic from 1/1/2015 to 1/1/2019. Patients with CD4+ T cell count < 200 cells/mm^3^, viral load >200 copies/mL, history of malignancy, or pregnancy were excluded. 870 patients met criteria. Patient demographics, lifestyle factors, medical co-morbidities, concurrent medications, and ART regimens were documented during the study period. The primary outcome was percent weight change over the follow up period. Secondary outcome was the odds of > 5kg weight gain over the study period. The effects of concurrent medications, medical comorbidities, ART combinations, and self-reported lifestyle behaviors on these outcomes were modeled using mixed effect linear and logistic regression analysis.

**Results:**

At baseline, 83.6% were male, 29.2% were African American, and 65.6% had a body mass index ≥ 25 kg/m. Over a mean follow up of 1.86 years, the study population gained a mean percent weight of 2.12 ± 0.21% (p< 0.001) with an odds of weight gain >5kg of 0.293 (p< 0.001). Male sex and increasing age were significantly associated with a decrease in percent weight over the study period as reflected in the table below. Diet was also significantly associated with a decrease in percent weight change over the study period of -1.99 ± 0.47 %, p= < 0.001 and a lower odds of > 5kg of weight gain (OR= 0.70, 95% CI= 0.50 – 0.97, p=0.03). In regression models, combination therapy with tenofovir alafenamide (TAF) and integrase strand transfer inhibitor (INSTI) containing regimens were significantly associated with an increase in percent weight over the study period. Other significant factors including demographics and ART regimens are noted in Table 1.

Table 1. Multivariable Regression Models*

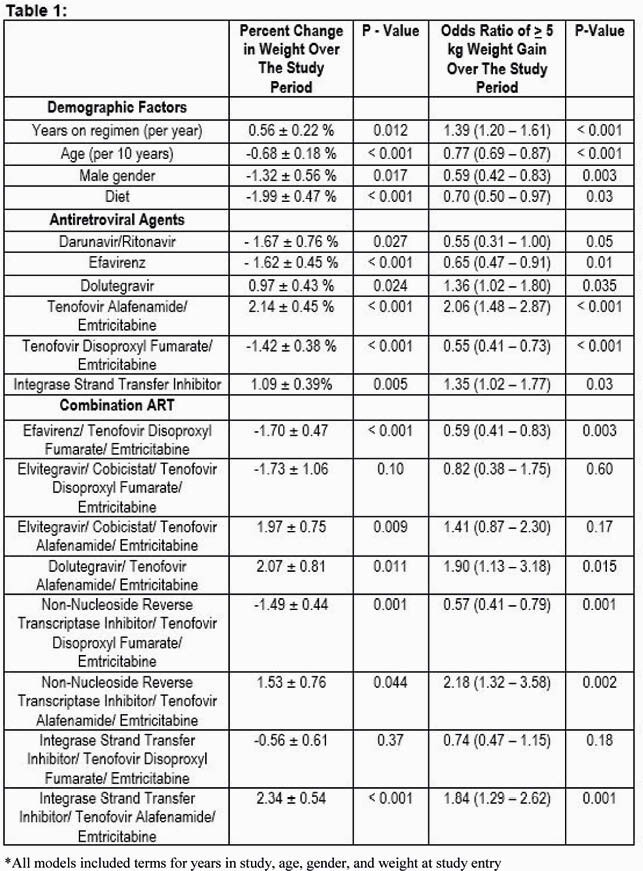

**Conclusion:**

Weight gain in PWH is multifactorial. Key factors associated with weight gain include combination therapy with TAF, particularly when combined with an INSTI. This data highlights the influential role of diet in PWH at risk of ART-associated weight gain.

**Disclosures:**

**Carlos Malvestutto, M.D.**, **Lilly** (Scientific Research Study Investigator)**Regeneron Inc.** (Scientific Research Study Investigator)**ViiV Healthcare** (Advisor or Review Panel member)

